# Accelerating Global Access for Indication Extension Through Regulatory Reliance Pilot: The First Indication Extension Pilot in Oncology

**DOI:** 10.1007/s43441-026-00984-2

**Published:** 2026-05-13

**Authors:** Anna Litsiou, Melanie Jane Snow, Emma Morgan, Priti Shah, Agathe Cabarrot, Rhiannon Davies

**Affiliations:** AstraZeneca UK, 136 Hills Road, Cambridge, CB2 8PA UK

**Keywords:** Regulatory reliance, Indication extension, Convergence, Transparency, Regulatory efficiency

## Abstract

Regulatory reliance pathways are increasingly being recognized as essential tools to accelerate patient access to medicines globally while building National Regulatory Authorities (NRA) capacity and capability. Although significant progress has been made in a number of international pilots for Chemistry, Manufacturing and Controls (CMC) post approval changes, there has been more limited application to labelling variations including indication extensions. This paper describes the first reliance pilot for an indication extension, which engaged 21 NRAs across multiple regions. The pilot leveraged the European Medicines Agency (EMA) as a reference agency and incorporated a digital platform, whose use was optional by the NRAs involved, for real-time sharing of questions and responses. The objectives were to reduce approval timelines, promote regulatory convergence, streamline health authority questions and enhance transparency among participating authorities. Results demonstrated significant reduction in approval timelines, with an average reduction of 3 months per country compared to standard timelines across participating countries. This article outlines the process of establishing the pilot, including planning and engagement with regulatory authorities, analysing success factors and challenges encountered including recommendations for optimising the process further. The findings suggest that regulatory reliance for indication extensions can substantially improve efficiency in regulatory processes, reducing approval timelines and ultimately benefiting a wider patient population to access medicines globally and timely.

## Introduction

Regulatory reliance is increasingly recognized as a key framework for improving global access to medicines while optimizing limited regulatory resources. The World Health Organization (WHO) defines regulatory reliance as “the act whereby a regulatory authority in one jurisdiction may take into account and give significant weight to assessments performed by another regulatory authority or trusted institution in reaching its own decision” [1]. Although progress has been made in implementing reliance pathways for CMC post-approval changes, experience with indication extensions remains limited. Indication extensions represent important modifications to existing approved products, often expanding their therapeutic indications or patient populations. Timely approval of indication extensions is crucial to ensure patients can benefit from new therapeutic applications of established medicines.

International efforts to streamline regulatory processes have gained momentum in recent years. The WHO's Good Reliance Practice (GRelP) Guideline [2] encourages regulatory collaboration throughout a product's lifecycle. The International Coalition of Medicines Regulatory Authorities (ICMRA) has developed frameworks for collaborative regulatory assessments. Industry associations have contributed through position papers addressing common challenges and promoting reliance pathways with harmonised dossier content, submission requirements and review timelines [3, 4].

Building on these initiatives, the first indication extension reliance pilot for an oncology treatment for advanced epidermal growth factor receptor (EGFR) mutated (EGFRm) NSCLC (Non-Small Cell Lung Cancer; Oncology Treatment hereafter) was initiated by AstraZeneca, leveraging the European Medicines Agency (EMA) as a reference agency and the support of World Health Organisation (WHO). This pilot aimed to test the application of reliance principles to expedite approvals for an indication extension across multiple jurisdictions, with a particular focus on emerging markets.

This article outlines the design, implementation, and outcomes of the first Oncology Treatment Reliance Pilot for an indication extension, providing insights into the practical application of reliance principles for indication extensions and the potential benefits for patients, regulators, and industry.

## Methods

### Case Study Selection Criteria

The Type II clinical indication extension reliance pilot was selected based on the following criteria:

1. The indication extension had high public health impact, expanding treatment options for patients
2. The reference country authority’s (EMA) approval was expected within a predictable timeframe, allowing sufficient time to engage stakeholders and NRAs globally
3. The EMA final unredacted assessment report would be available for sharing with participating authorities
4. The product is registered in multiple countries, providing a diverse geographical representation for the pilot

Based on these criteria, an oncology treatment was selected for the pilot. The treatment is an epidermal growth factor receptor (EGFR) tyrosine kinase inhibitor indicated for the treatment of NSCLC [5]. The indication extension, based on the data of the Phase III Study, represented an important expansion of the therapeutic indication for the specific treatment [5].

### Criteria for the Selection of Countries

The pilot targeted countries across multiple regions where the oncology treatment for NSCLC was already registered and the indication extension into a new population would be submitted. The selection criteria for participating countries included:

1. Countries with established regulatory frameworks but potentially lengthy review timelines for indication extensions
2. Countries that had expressed interest in implementing reliance pathways
3. Countries representing diverse geographical regions and regulatory systems (varying data requirements and submission formats e.g. CTD/eCTD)
4. Countries with varying levels of regulatory maturity for indication extensions (different classifications for indication extensions, different approaches to the assessment of benefit-risk profiles)
5. Countries with variable implementation periods after regulatory approval

Based on these criteria, 21 countries were invited to participate in the pilot. These countries represented diverse regions including Asia–Pacific, Middle East, Africa, and Latin America.

### Pilot Description and Objective

In this reliance pilot, participating NRAs had the opportunity to apply reliance by leveraging the EMA assessment and approval to reduce their review timelines. The participating NRAs could decide, upon receipt of the EMA final unredacted assessment report and approval, if they would fully or partially rely on the EMA evaluation in reaching their own independent decision.

The objectives of the reliance pilot included:

- Reducing approval timelines for the indication extension, targeting a reduction of at least 30% compared to standard timelines
- Enhancing transparency among regulatory authorities through the sharing of a reliance package consisting of the EMA assessment report and EMA questions and answers (Q&As), which were also shared on the digital platform.
- Testing the utility of a digital platform for real-time sharing of regulatory questions and responses raised by the other participating NRAs in the pilot. The utility was optional to assure NRA discretion and flexibility.

To participate in the pilot, the following prerequisites were established:

- Adherence to proposed reduced review timelines
- Acceptance of EMA as a reference agency
- Willingness to follow EMA submission standards and avoid requesting country-specific documents or data where possible
- Optional participation in a digital platform for real-time sharing of questions and responses, involving minimal training for the NRAs.

### Overall Process and Timeline

The pilot involved three phases:

1. *Planning phase*: Project screening criteria were defined, the product was selected, and the regulatory strategy was developed for each target country.
2. *Engagement phase*: During this phase, the sponsor approached EMA, WHO and the participating NRAs to introduce the pilot concept and secure their participation.
3. *Execution phase*: Following EMA's approval of the indication extension, the sponsor-initiated submissions to the participating NRAs nationally, shared the reliance package (EMA final assessment report, EMA approval letter and EMA Q&As) with the submitted dossier and on a digital platform simultaneously, and tracked the review process and approval timelines for each NRA.

**Fig. 1:**
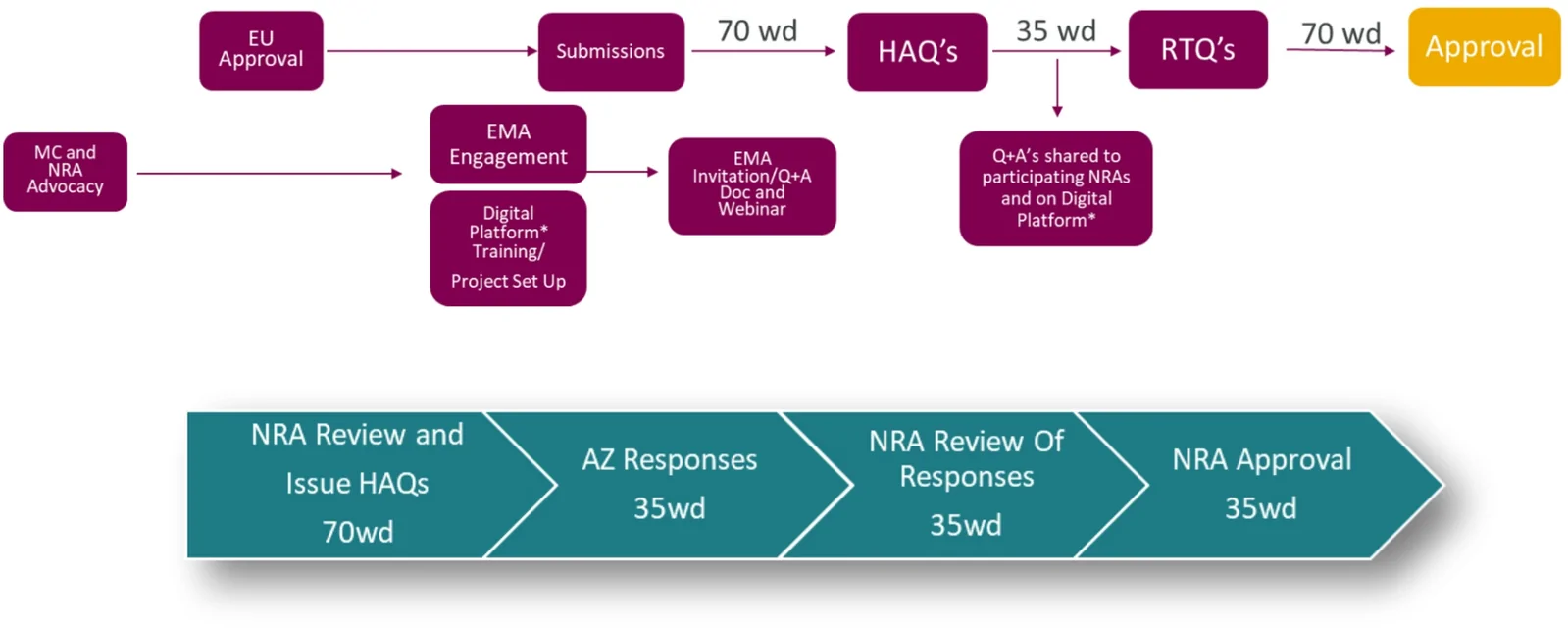
Process flow and proposed timelines for the indication extension pilot. Advocacy with NRA and EMA was completed ahead of the submissions. Participating NRAs were asked to perform the reviews using the timeline above. *The provision of the digital platform was by a third party

The pilot established a target timeline guideline for participating NRAs (see Fig. 1):

- 7 days for submission of the dossier
- 70 working days allocated for NRAs to review and raise questions
- 35 working days for AstraZeneca to respond
- 35 working days for NRAs to review responses
- 35 working days to grant approval

This resulted in a total target timeline of 8 months from submission to approval, which represented a significant reduction compared to an average standard timeline of 12 months for the participating countries. The initial targeted timeline presented to the NRA’s was 6 months, but was slightly extended by two months during the pilot to allow for flexibility. Some authorities may have shorter timelines that those set up for this pilot, and those NRA’s were asked to maintain their standard review times at the minimum.

## Results

### Planning Phase

The pilot planning phase began with defining selection criteria for both the product and target countries in first half of 2024. After selecting the indication extension for the oncology treatment based on the Phase III clinical study read-out, a taskforce was formed to develop a detailed pilot roadmap and engagement plan.

The team decided to propose EMA as the reference agency considering the EMA’s strong commitment to international collaboration and reliance, and the availability of its detailed final assessment reports, for which the unredacted versions of final assessment were provided through the NRA Submission Pathways and the digital platform. The team also developed a comprehensive global filing plan to assess the impact on internal resources and to ensure alignment with the company's global regulatory strategy for the specific oncology treatment. The pilot initiative was communicated to EMA and WHO Teams.

### Engagement Phase

#### Engagement with NRAs

From mid-2024, the sponsor affiliates, contacted NRAs in 21 countries to present the pilot concept. An introduction package was submitted to all NRAs, including:

- A presentation on the reliance pilot concept and objectives
- A Q&A document outlining the background and addressing critical aspects of the pilot
- Information about the optional digital platform for real-time sharing of questions and responses
- As this was the first in industry indication extension pilot, an EMA written invitation was also provided.

The NRAs were also invited to an introductory webinar, hosted by AZ, with critical attendance from both EMA and WHO alongside the Sponsor, where the pilot's scope was introduced. The participation of EMA and WHO was instrumental, as their presence enabled interested and participating NRAs to raise questions and concerns directly with these key regulatory authorities.

Of the 21 NRAs approached:

- 13 (61.9%) confirmed their participation in the pilot
- 6 (28.6%) received the reliance package but did not confirm to formally join the pilot
- 1 (4.8%) declined to participate
- 1 (4.8%) did not respond and did not receive the reliance package

**Fig. 2:**
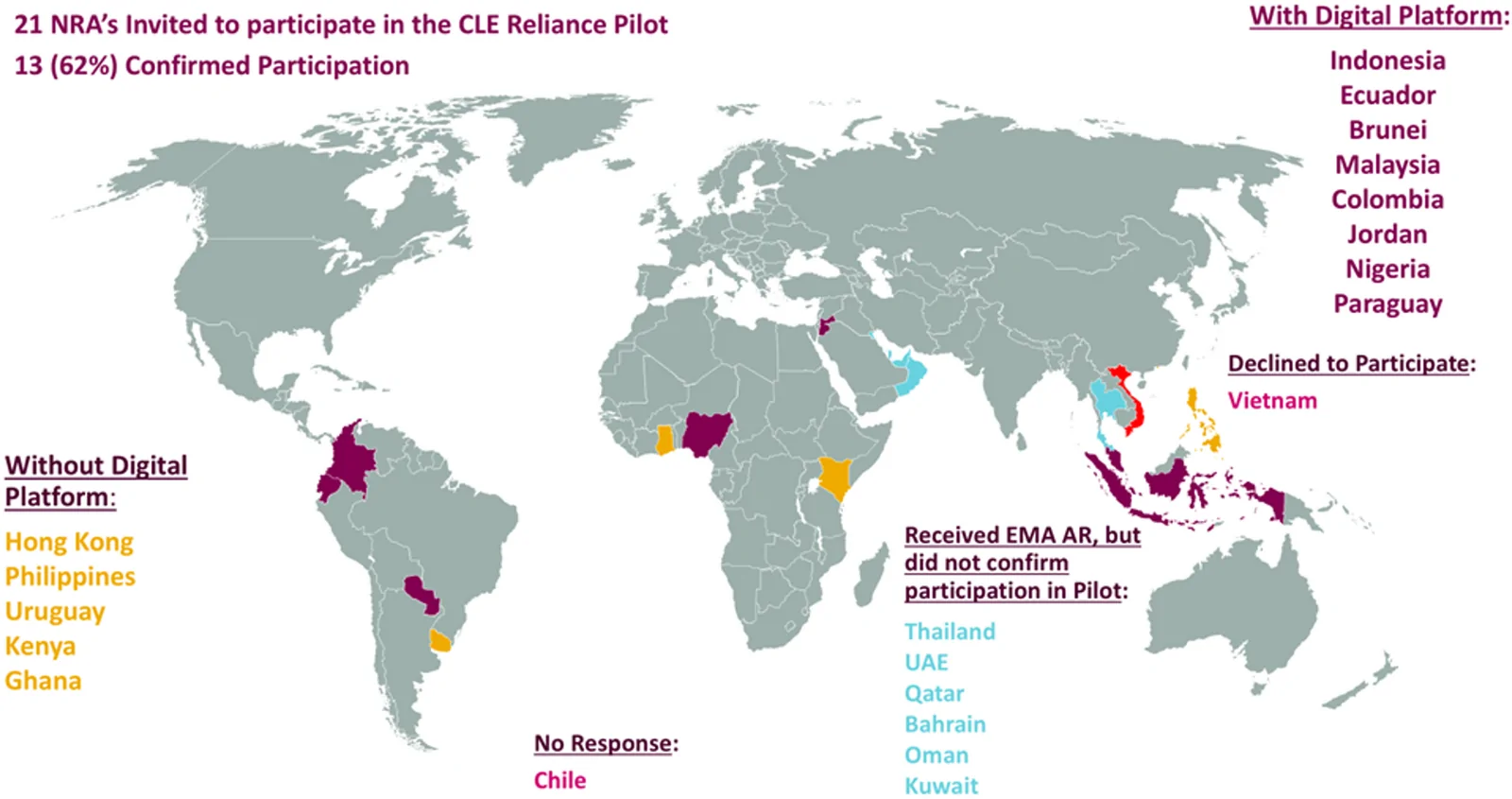
Worldwide map of countries whose NRAs were invited to participate in the pilot

Among the 13 confirmed participants, 8 (61%) agreed to training and access to the digital platform for access to the EMA reliance package and to real-time sharing of participating NRA questions and responses. The remaining five NRAs preferred to follow their standard communication channels. See Fig. 2 for a visual representation of the global NRA participation.

#### NRA Feedback—Reliance Participation

Of the NRA’s who responded to our pilot invitation, the following reasons for not participating were provided:

- No legal or regulatory pathway exists for reliance
- NRA resource constraints
- Can get faster approvals through local pathways

NRA Feedback—Use of Online Platform.

- 3 out of 8 NRA’s (37%) wanted to use online platform as observer only.
- NRA’s raised questions about
  - Ensuring continuous access to and archiving of the documents provided on the platform.
  - The long-term plan for managing and maintaining the platform, including funding support for the platform itself.

### Success Factors

#### Transparency and Trust

Transparency was a key factor in establishing trust between the sponsor and the participating regulatory authorities. The sponsor committed to sharing the unredacted and final EMA assessment report (personal data were redacted as per EU GDPR regulations and EU policy 70) [6, 7] and all questions and answers from the EMA review process with participating NRAs. This transparency enabled NRAs to understand the depth and rigor of the EMA's assessment, facilitating their review decision adopting good reliance practices.

#### Digital Platform for Real-Time Sharing

The digital cloud platform, with security controls and CCI protection, provided a novel approach to enhance transparency and efficiency in the regulatory review process. The platform allowed for real-time sharing of questions from any participating authority and the company's responses, enabling all participants to benefit from the collective regulatory dialogue. This approach aimed to avoid duplication of questions and facilitate a more efficient review process.

In addition, Q&As raised by other participating NRAs were shared through the traditional communication channels enhancing transparency and trust with those NRAs not using the digital platform.

#### Product Sameness

According to WHO, assuring sameness of product is essential for the use of reliance [8]. In this pilot, the same clinical data package that supported the EMA approval was submitted to all participating NRAs, ensuring consistency in the benefit-risk assessment across jurisdictions.

### Execution Phase

#### Submissions Achieved Within Target Timeframe

All submissions to participating NRAs were completed within a three-week window, by early October 2024. This synchronized approach allowed for a coordinated implementation of the pilot across all participating countries. Worth noting that the reliance package was shared on the digital platform for a simultaneous access to the submission dossier submitted through each NRA national pathway for indication extension.

#### Regulatory Review Status

At the end of the pilot (May 2025), 15 of the 19 countries that received the reliance package had approved the indication extension. Of the 4 remaining countries, 2 are now approved and 2 remain under review at the time of publication (See Table 1).

**Table 1:** Time saved per country during the pilot

Countries	Timelines (Actual vs. Standard)	Time saved
Oman^1^	1 week vs. standard 8 months	~ 7.8 months
Uruguay	1.5 months vs. standard 6 months	4.5 months
Thailand^1^	3 months vs. standard 12 months	9 months
Bahrain^1^	1.5 months vs. standard 9 months	7.5 months
Jordan	2.25 months vs. standard 6 months	3.75 months
Ecuador	2 months vs. standard 6 months	4 months
Kuwait^1^	4 months vs. standard 8 months	4 months
Paraguay	4 months vs. standard 6 months	2 months
Qatar^1^	3.75 months vs. standard 6 months	2.25 months
UAE^1^	1.5 weeks vs. standard 3 months	2.5 months
Malaysia	5 months vs. standard 7 months	2 months
Hong Kong	6 months vs. standard 9 months	3 months
Nigeria	7 months vs. 3 months	0 months
Kenya	7.25 months vs. standard 12 months	4.75 months
Indonesia	7.75 months vs. standard 15 months	7.25 months
Brunei	8.5 months vs. 15 months	6.5 months
Colombia	24 months vs. 30 months	6 months
Ghana	Under Review	NA
Philippines	Under Review	NA

^1 =^ Countries who received reliance documents but did not confirm participation in the pilot. Time savings may not be attributable to the pilot

#### Health Authority Questions

The number of questions received from participating NRAs (6 HAQs in total) was slightly lower than typically experienced for indication extensions (the previous clinical indication extension, for the same disease, experienced 9 HAQs from the participating NRAs). Several countries (Uruguay, Jordan, Paraguay, Hong Kong, Nigeria, Kenya, Indonesia) approved the application without any questions, suggesting full reliance on and agreement with the EMA assessment.

Other countries (Ecuador, Malaysia and Philippines) raised limited questions, primarily focused on local labelling requirements or administrative aspects rather than scientific or clinical concerns. Of note, Philippine FDA requested the local label to be fully aligned to the EU SmPC, but the reference label used in Philippines is the company core data sheet (CCDS). Only Colombia requested further data for overall survival, frequently requested for other oncology treatments under review based on industry experience. This reduction in questions demonstrated the value of the reliance approach in streamlining the review process.

#### Reliance Metrics

**Fig. 3:**
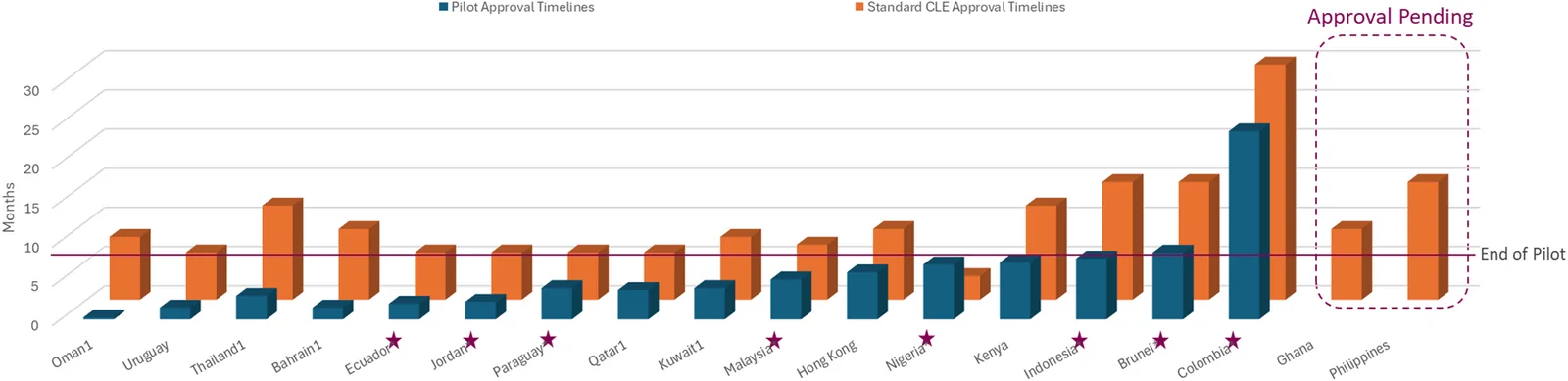
Pilot actual approvals.  NRA’s who agreed to use a digital platform. “Country Name followed by number 1” = NRA’s who received the reliance package, but did not confirm participation (not included in metrics)

The pilot demonstrated significant reduction in approval timelines for those NRAs participating in the pilot, including countries with shorter review timelines compared to the pilot timelines (See Fig. 3):

- For all 13 NRAs receiving the reliance package:
  - The average *standard* approval time was 12 months
  - The average *pilot* approval time for all 13 participating NRAs was 7.7 months
  - The average time saving for the approval was 4.5 months (38% reduction)
- For the 9 participating NRAs in the pilot who approved within the pilot timeframes:
  - the average *standard* approval time was 7.8 months
  - The average *pilot* approval time was 4.8 months,
  - the average time saving was 3.0 months (38% reduction)

One country (Nigeria) did not experience timeline reduction due to ongoing audits at the NRA that delayed all their drug applications. This demonstrates that external, agency-level processes are beyond the sponsors’ control and can constrain timelines irrespective of pilot design. Approvals are awaited for Philippines, who have seen delays in all reviews at the FDA and Ghana, where the health authority is moving all applications to a digital portal which has created a stock-pile of a lot of physical applications.

#### Implementation Barriers and Systemic Challenges

The pilot phase revealed valuable insights into implementing reliance-based regulatory pathways for an indication extension for an oncology treatment, highlighting key opportunities to enhance and optimize future initiatives for wider patient population access for labelling updates while building on the promising benefits already demonstrated through the pilot as the regulatory environments of the NRAs evolve through these pilots’ facilitating reliance.

#### Flexible Implementation Models Reflecting Regulatory Maturity

Although all participating NRAs embraced the reliance concept, the pilot implementation approach varied across jurisdictions, reflecting different stages of regulatory maturity, legal frameworks, and national risk assessment priorities. While most authorities relied on the EMA assessment reports as the main scientific basis rather than doing a full independent assessment, some approved the applications without additional queries while others raised a number of additional requests.

However, to optimize the efficiency and attractiveness of reliance pathways for industry sponsors, enhanced transparency and predictability are essential. Sponsors would benefit from clear upfront communication regarding the anticipated scope of reliance types (recognition, verification, or abridged review) that each authority will apply and more standardized, predictable timelines for regulatory decisions across participating jurisdictions. Establishing transparent regulatory pathways that define reliance levels (e.g. recognition, verification, or abridged review) and associated timelines would provide greater certainty for applicants while respecting the legitimate regulatory sovereignty of participating authorities.

#### Digital Platform Adoption and System Integration

The optional digital platform for real-time sharing of the reliance package, and questions and responses raised by the other NRAs in the pilot was adopted by 8 of the 13 confirmed participants. Three countries (Brunei, Malaysia, Colombia) opted to join as "observers," accessing the shared information but continuing to use the standard communication channels for their own questions. A significant implementation challenge emerged from the lack of integration between the collaborative platform and national regulatory systems, ie. standard pathway defined by the NRA legislation, requiring the sponsor to submit the applications through both channels—a duplication of effort and resource-intensive but necessary to adhere to jurisdictional legal requirements for submission. This experience highlighted the need for both flexibility in implementing new tools within established regulatory processes and technical solutions that enable seamless connectivity between collaborative and national platforms to eliminate redundant submissions and streamline the application process.

#### Legal and Procedural Constraints

Participating NRAs encountered different legal and procedural constraints that limited their ability to fully implement reliance mechanisms or participate in the pilot:

1. *Statutory Assessment Requirements*: Mandatory local review frameworks for indication extension through local clinical commissions, external to the NRAs, or external clinical reviewers to evaluate independently illustrates how national regulatory evaluation frameworks can differ; and how such frameworks require adaptation to facilitate reliance review practices, including reduced review timelines.. Understanding the NRA ecosystem of stakeholders involved in the dossier review is key for future adoption of good regulatory reliance review practice.
2. *Administrative Documentation Barriers*: Legal requirements for Certificates of Pharmaceutical Product (CPP) and document legalization added additional administrative complexity and time to the submission process, even when EMA Approval Letter and Assessment Report were available and may be deemed sufficient as proof of Reference Agency’s official review and approval.
3. *Dual Submission Inefficiencies*: Applications had to be submitted through both: national pathway digital channels and the collaborative platform for participating NRAs, requiring sponsors to manually upload documents multiple times—a cumbersome and resource-intensive process that undermined efficiency gains. In addition, the submissions in the cloud platform required no eCTD validation and were presented as a ‘PDF’ that could easily be navigated while requiring security and validation checks by the sponsor
4. *Inflexible National Timelines*: Mandatory review timelines established in national legislation and non-waivable procedural requirements constrained flexibility in adopting collaborative review schedules.
5. *Resistance from High-Capacity Authorities*: Some countries with already expedited national review processes were initially resistant to participate, as their existing timelines were faster than the proposed collaborative pathway, highlighting the need to better articulate and demonstrate the broader advantages of multicounty reviews beyond timeline efficiency—including enhanced regulatory capacity through shared expertise, improved scientific rigor, reduced duplication of effort, and improved patient access to medicines across participating jurisdictions.
6. *Variability in Dossier Requirements*: Different regulatory packages were needed for different countries, particularly when safety-related changes were bundled for some markets but not others. This is another example of how diverse the NRA Post Approval Frameworks can be presenting opportunities or challenges like the bundling of safety changes with some NRAs.
7. *Market-Specific Packaging Constraints*: Countries with shared packs but differing grace periods for implementation posed challenges for inclusion in the pilot, as the sponsor needed adequate lead time to align production and distribution across multiple markets especially with faster approval times but different implementation times.
8. *Resistance to Adoption of Digital Platforms*
  Regulatory authorities often hesitate to adopt digital platforms for reliance initiatives due to concerns about data security, continuous access, and potential conflicts of interest. These concerns can be addressed by ensuring regulators retain authority over the official record and maintain independent access to all documentation through traditional business processes, whilst providing platform-based continuous access during active projects and read-only access thereafter, with full download capabilities for archival purposes. Innovative digital capabilities should strengthen data integrity through immutable versioning and comprehensive audit trails, and cybersecurity risk should be equal to or lower than current systems through stronger centralized controls. Transparent governance, clear ownership boundaries that prevent sponsors from exerting undue control, and the financial stability of the platform provider together enhance transparency, traceability, and oversight, aligning with WHO Good Reliance Practices (2021) and modern regulatory expectations. Taken together, these measures demonstrate that digital platforms can overcome initial resistance by preserving regulatory independence whilst materially improving collaboration efficiency.

## Discussion

This Reliance Pilot for a new indication extension demonstrated the potential of regulatory reliance to significantly reduce approval timelines for indication extensions across multiple jurisdictions. The average reduction in approval time of three months (38% reduction) represents a substantial improvement in efficiency and translate to earlier patient access to important new therapeutic options.

Key lessons emerged from this pilot:

1. *Transparency builds trust*: Sharing the complete EMA assessment report and Q&As with participating NRAs was crucial in establishing the trust necessary for effective reliance. This transparency enabled NRAs to understand the depth and rigor of the reference agency's assessment, facilitating their own decision-making process. Transparency is also a lot faster with Technology Cloud Platforms.
2. *Digital tools enhance collaboration*: The optional digital platform for real-time sharing of questions and responses demonstrated the potential of technology to enhance regulatory collaboration by improving transparency and providing one stop viewing of submissions as a PDF.
  By enabling all participating authorities to see questions raised by others and the company's responses, the platform also helped to avoid duplication and promote consistency in regulatory assessments.
3. *Flexibility is essential*: The pilot's flexible approach, allowing NRAs to determine their level of participation and engagement with the digital platform, was important in securing broad participation. This flexibility acknowledged the different regulatory frameworks, resources, and comfort levels with reliance among participating authorities.
4. *Reliance does not mean removal of ability to make sovereign decisions by NRAs*: All participating NRAs maintained their regulatory sovereignty and made independent decisions based on their assessment of the available information. Reliance enabled them to focus their potentially limited resources on aspects most relevant to their specific context, while leveraging the detailed assessment conducted by EMA and its applicability in their country.
5. *External factors can impact timelines*: The experience in Nigeria, where ongoing audits delayed the approval despite participation in the reliance pilot, highlighted that external factors can impact regulatory timelines even with well-designed reliance initiatives.
6. *Aligned and near-concurrent submissions of efficacy and safety changes*: submission at this pace allows faster and coordinated regulatory reviews across regions, and reducing delays in patient access to improved and innovative treatments. This ensures patients benefit sooner from updated evidence on a medicine’s effectiveness and safety profile, promoting consistent global care standards.

The pilot also identified several areas for further development and improvement:

1. *Legal and procedural frameworks*: Some NRAs faced legal or procedural constraints that limited their ability to fully implement reliance. Ongoing regulatory reform is needed to address these constraints and create more enabling frameworks for reliance. For example, as experience is gained through the pilots like the one presented in this paper, NRA regulatory systems can adapt to include reliance pathways in such a legal form entailing optimised timelines to try and increase predictability of procedures and optimisation of resource utilisation.
  Also, another future adaptation of reliance pathways is reassessing the need for country specific and legally required documents like the CPP as the provision of the EMA assessment report and Q&As may make the need for this document and other country specific documents duplicative. Reviewing the legal framework for the need for these documents may further streamline and optimise the process.
2. *Capacity building*: Building processes and frameworks within NRAs to effectively implement reliance approaches remains important. This includes developing skills in critically assessing reference agency evaluations and adapting them to local contexts.
3. *Standardization of reliance pathways*: Greater standardization of reliance pathways across countries would facilitate more efficient implementation of global reliance strategies. This could include harmonized documentation requirements, review timelines, and communication processes.
4. *Expansion to other product types and changes*: The success of this pilot suggests that similar approaches could be applied to other types of products and regulatory changes, including complex biologics, advance therapeutics, vaccines, and manufacturing changes.

## Conclusion

This first indication extension reliance pilot for an oncology treatment demonstrated that regulatory reliance can significantly reduce approval timelines for indication extensions across multiple jurisdictions. The average reduction in approval time of three months (38% reduction from expected) represents a substantial improvement in efficiency and could translate to earlier patient access to important new therapeutic options.

The pilot highlighted the importance of transparency, flexibility, and collaboration in implementing effective reliance pathways. It also identified areas for further development, including legal and procedural reforms, capacity building, and standardization of reliance approaches.

As regulatory systems continue to evolve in response to increasing complexity and resource constraints, reliance offers a promising approach to enhance efficiency while maintaining high standards of regulatory oversight. By building on the lessons learned from this pilot and similar initiatives [9–12], regulatory authorities and industry can work together to develop more effective reliance pathways that ultimately benefit patients through more timely access to medicines.

## Data Availability

All data supporting the findings of this study are available within the paper.
